# Galaxy CloudMan: delivering cloud compute clusters

**DOI:** 10.1186/1471-2105-11-S12-S4

**Published:** 2010-12-21

**Authors:** Enis Afgan, Dannon Baker, Nate Coraor, Brad Chapman, Anton Nekrutenko, James Taylor

**Affiliations:** 1 Department of Biology and Department of Mathematics & Computer Science, Emory University, Atlanta, GA 30322, USA; 2 Huck Institute for the Life Sciences, Penn State University, University Park, PA 16802, USA; 3 Department of Molecular Biology, Simches Research Center, Massachusetts General Hospital, Boston, MA 02114, USA

## Abstract

**Background:**

Widespread adoption of high-throughput sequencing has greatly increased the scale and sophistication of computational infrastructure needed to perform genomic research. An alternative to building and maintaining local infrastructure is “cloud computing”, which, in principle, offers on demand access to flexible computational infrastructure. However, cloud computing resources are not yet suitable for immediate “as is” use by experimental biologists.

**Results:**

We present a cloud resource management system that makes it possible for individual researchers to compose and control an arbitrarily sized compute cluster on Amazon’s EC2 cloud infrastructure without any informatics requirements. Within this system, an entire suite of biological tools packaged by the NERC Bio-Linux team (http://nebc.nerc.ac.uk/tools/bio-linux) is available for immediate consumption. The provided solution makes it possible, using only a web browser, to create a completely configured compute cluster ready to perform analysis in less than five minutes. Moreover, we provide an automated method for building custom deployments of cloud resources. This approach promotes reproducibility of results and, if desired, allows individuals and labs to add or customize an otherwise available cloud system to better meet their needs.

**Conclusions:**

The expected knowledge and associated effort with deploying a compute cluster in the Amazon EC2 cloud is not trivial. The solution presented in this paper eliminates these barriers, making it possible for researchers to deploy exactly the amount of computing power they need, combined with a wealth of existing analysis software, to handle the ongoing data deluge.

## Background

Widespread availability of high-throughput DNA sequencing instruments, and the development of novel techniques based on sequencing, provides a potentially very valuable resource for researchers in genomics. However, transforming sequence data into biologically meaningful information requires sophisticated computational infrastructure and support. The size of the required computational infrastructure is outpacing what many labs and even universities are able to support. In addition, the setup and maintenance associated with a computational infrastructure presents significant problems for individual investigators and small labs that may not have the necessary informatics support.

Fortunately, a computational model – cloud computing [1] – has recently emerged and is ideally suited for the analysis of large-scale sequence data. In this model, computation and storage exist as virtual resources in remote datacenters, and can be dynamically allocated and released as needed. However, cloud computing resources are not yet suitable for immediate “as is” use by experimental biologists. In the current model, cloud resources are acquired as independent, stripped-down units that must first be customized for the intended use. They then must be configured to work in unison and a mechanism must be provided for the data uploaded and analyzed on those resources to persist beyond the life of a (usually transient) cloud compute resource.

To date, there are several projects and solutions in the context of high throughput sequencing and bioinformatics in general that utilize cloud computing to deliver the computational capacity and on-demand scalability (e.g., Crossbow [2], CloudBurst [3], RSD-cloud [4], Myrna [5]). However, these projects primarily target specific problems and provide custom solutions for a given tool or methodology. Although very valuable, such tools provide minimal help for researchers wanting to compose a variety of tools into an analysis pipeline or for researchers that want to use their own tools when utilizing cloud computing resources. Thus, there is a need for a flexible solution that can be utilized in a variety of scenarios and that provides support for customization.

This paper introduces CloudMan from the Galaxy Project [6], an integrated solution that leverages existing tools and packages by providing a generic method for utilizing those tools on cloud resources and abstracting out low-level informatics details. This solution handles all of the intricacies of cloud computing resource acquisition, configuration, and scaling to deliver a personal compute cluster in a matter of minutes. All interaction with Galaxy CloudMan and the associated cloud cluster management is performed through a web based user interface and requires no computational expertise. The deployed cluster comes preconfigured with all of the bioinformatics packages available in the NERC Bio-Linux workstation (version 6) [7] as well as a range of NGS tools available within the Galaxy framework [8]. Moreover, the process of tool deployment is fully automated and decoupled from the base machine image, making it possible to very simply add additional tools to an individual cloud cluster instantiation.

## Results

### Instantiating and controlling a cloud cluster

The Galaxy CloudMan application currently supports creation of a compute cluster on Amazon’s EC2 [9] cloud computing infrastructure. The process of instantiating a cluster does not require any computational experience, and requires no compute infrastructure or software beyond the web browser used to control the cluster. Galaxy CloudMan is thus ideal for independent researchers and small labs that have a specific or periodic need for computational resources but lack informatics expertise and commitment to manage and maintain a computational cluster. The process of instantiating a CloudMan compute cluster consists of three steps: (1) create an Amazon Web Services (AWS) account and sign up for the EC2 and S3 services, (2) use the AWS Management Console to start a master EC2 instance, and (3) use the CloudMan web console on the master instance to manage the cluster size. Step one needs to be performed only once, usually by a person controlling the cloud cluster. Steps two and three need to be performed each time running jobs on a compute cluster is desired, but, again, only by the person controlling the cluster. Once set up, additional users may use the cluster simply through the Galaxy web interface without requiring any system accounts or privileges. A single instance of CloudMan controls a single cluster – of potentially variable size – but a single user may create as many CloudMan cluster instances as desired.

Once CloudMan starts, it automatically configures the master instance as a head node of a Sun Grid Engine (SGE) [10] compute cluster but it does not start any additional worker instances or assign persistent storage to the cluster. In the context of cloud computing, compute instances are usually transient, meaning that any changes made to an instance while the instance is alive are lost at instance termination. In order to persist any data uploaded to the cloud or any analysis results, the data needs to be stored on an external data volume. In the case of CloudMan on EC2, Amazon’s Elastic Block Storage (EBS) [11] volumes are used for data persistence.

**Figure 1:**
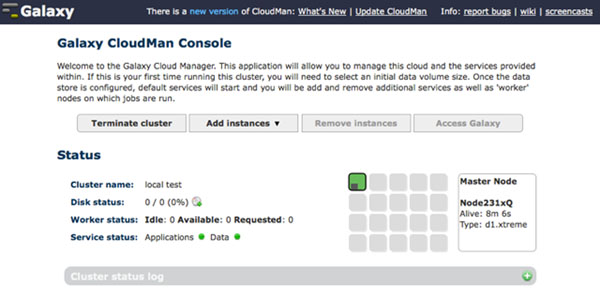
**Main web interface for Galaxy CloudMan.** Screenshot of the CloudMan cloud controller web interface running on the master instance of the cloud compute cluster. This interface is used to control the size of the cloud cluster, including adding cluster services, scaling the size of the cluster in terms of worker instances and associated persistent data volume, and as an overview of the cluster status.

Once available, the CloudMan web interface (Figure 1) allows a user to configure additional features of the cluster. Currently, the following features are supported: association of a persistent data volume with the cluster, addition of a range of NGS tools (see below), and addition of the Galaxy analysis interface. Without a persistent data volume, a user may use the cluster for a proof-of-concept computation or a one-time analysis. For clusters that are maintained over time, adding persistent storage is initiated with a click of a mouse, with all infrastructure intricacies handled automatically by CloudMan. Similarly, if a completely configured instance of Galaxy is desired for use of a range of NGS tools, it is trivial to do so through the CloudMan UI.

**Figure 2:**
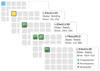
**Scaling worker instances within CloudMan**. A progression of the act of scaling the number of worker instances associated with the given cloud cluster. Each icon represents an individual cloud instance. Within each icon, the load of the instance over the past 15 minutes is shown as a small glyph. Based on the load of worker instances, the user can decide to scale the size of the cluster up or down.

In addition to the user-level cluster functionality, CloudMan makes it easy to exploit what is arguably the most unique and powerful feature of cloud computing - elasticity. Through the CloudMan web interface, one can scale the size of the cloud cluster at runtime by adding or removing worker instances comprising the cluster (Figure 2). Similarly, the size of the persistent data volume (i.e., EBS volume) associated with a cluster can easily be expanded. Within EC2, individual EBS volumes used as persistent data storage medium within CloudMan, have a predefined size. As the use of a given cluster expands, users may consume the space associated with the given cluster. The CloudMan web interface allows ‘growing’ the size of the persistent data volume associated with a cluster. In the background CloudMan orchestrates the following steps to accomplish the task at hand: (1) stop any services using the user data volume, (2) detach the current user data volume from the master instance, (3) snapshot the detached volume, (4) create a new volume of user-specified size based on the snapshot from step 3 and attach it to the master instance, (5) grow the file system on the new data volume, and (6) resume any services.

Because Galaxy CloudMan is built on top of a Bio-Linux machine image, all of the tools available within Bio-Linux can be used on the instantiated cloud cluster. Accessing the Bio-Linux tools is realized through a command line interface - just like on any other compute cluster. As indicated earlier, the SGE job manager is configured and used on the cluster, making it possible for users to simply copy their job scripts to the cloud cluster and run them there - but with the scalability offered through cloud computing.

When a given cluster is no longer needed, the CloudMan web interface is used to terminate all of the services and worker instances. If persistent data storage was associated with the cluster, the data is preserved while the cluster is offline, and made available in the same state once the cluster is instantiated again. It takes only a few minutes to scale up or down a cluster and consume the required amount of resources.

### Tool availability

By default, the Galaxy CloudMan is built on top of a Bio-Linux machine image available from CloudBioLinux [12] and thus makes all of the tools packaged by NERC Bio-Linux [13] immediately available. NERC Bio-Linux represents a set of packaged and fully featured bioinformatics tools that enable users to focus on tool usage rather than tool installation and configuration. By building on top of such varied set of bioinformatics tools, one can combine the cluster controlling functionality of CloudMan with the variety of tools. In addition to the tools available through Bio-Linux, a set of NGS tools available through Galaxy are also available for use, including: Bowtie [14], BWA [15], and SAMtools [16]. If a user desires additional tools, we have provided a mechanism for streamlining the tool installation process (see Methods section). A script used to automatically install all the tools available to a default instance of CloudMan cluster is available at https://bitbucket.org/afgane/mi-deployment/; using this script and customizing it to include the desired tools provides a simple method for modifying the capabilities of a cluster instance. The script supports the ability to install additional tools at cluster runtime only or to persist the changes for future cluster invocations.

## Conclusions

To keep up with the growth of data being produced in life sciences and the accompanying computational demand, there is a need for increased access to computational resources. Cloud computing offers access to such resources but still makes it difficult to create complex deployments of useful standalone infrastructures. This is especially cumbersome for individuals and small labs that lack informatics support to fully harness this general-purpose infrastructure.

The default version of Galaxy CloudMan can be used “as-is” to support creation and control of fully functional compute clusters on cloud resources; it supports a broad range of bioinformatics tools and it makes it possible to add additional tools with little effort. The process of tool deployment is completely automated and well documented, making it reproducible in other environments. Overall, the system is simple to use and it targets individual researchers so they can gain access to the computational resources they need without requiring support from skilled bioinformatics personnel.

The source code for the entire project is available under the MIT licence and is available from http://bitbucket.org/galaxy/cloudman/. Documentation and detailed instructions on how to use Galaxy CloudMan are also available from http://usegalaxy.org/cloud.

## Methods

To deliver a ready-to-use compute infrastructure to individual researchers, we decided to build CloudMan as a modular framework that can be used “as is” but also supports easy customization. In keeping with this goal, we have embedded Galaxy CloudMan on top of the Bio-Linux workstation machine image and integrated it with Galaxy. This delivers a broad range of bioinformatics tools directly to CloudMan users.

**Figure 3:**
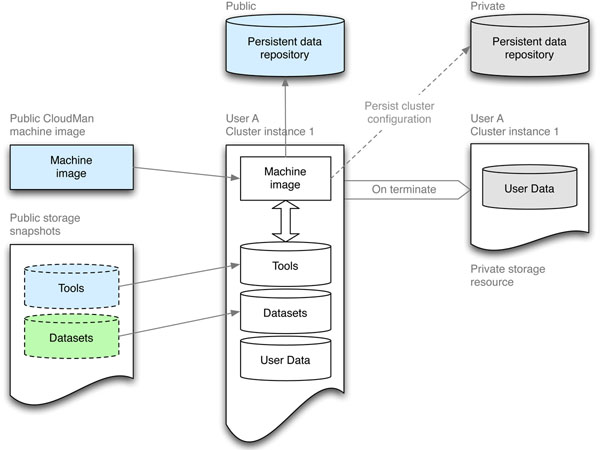
**Modular architecture of CloudMan**. The architecture of CloudMan is based on separation and subsequent coordination of otherwise independent components: the machine image, a persistent data repository, and persistent storage resources (i.e., snapshots). The *machine image* is characterized by simplicity; it consists only of the basic services required to initiate the application unit deployment process. The *persistent data repository* lives independent of the machine image and is used to provide instance contextualization details, such as, boot time scripts that define which services should be started. Lastly, *persistent storage resources or snapshots* are used as the storage medium for tools, libraries, or datasets required by the tools. Once instantiated, those components are aggregated by CloudMan into a cohesive operational unit. Because they are not modified during the life of a cluster, the persistent storage resources are deleted upon cluster termination. All of the cluster settings and user data are preserved in user’s account and will be reused on next cluster instantiation. The Galaxy CloudMan developers maintain items in blue; items in green are (currently) maintained by the CloudBioLinux community (http://www.cloudbiolinux.com/), while items in gray are private to a user.

To support the evolution of the tools and the project as a whole but minimize disruption of end user’s routine, the Galaxy CloudMan has been built with the modularity of the cloud infrastructure in mind. Specifically, we have the domain specific tools separate from the core components that enable CloudMan to operate. In the context of EC2, this is achieved by having a “thin” machine image that contains only the essential bootstrapping scripts and a well-defined instance boot process, while the desired tools reside on external data volumes (i.e., EBS volumes). Such an approach enables the same machine image to be used for a variety of scenarios and to evolve over time because it can be contextualized at runtime (i.e., prepare and deploy an otherwise generic instance) [17]. This model enables us to update the functionality of CloudMan without requiring the users to alter their routine; they continue to use the infrastructure as they are accustomed to and still have the option of updating the functionality offered by CloudMan (Figure 3).

By having domain specific tools on external data volumes, it is easy to modify the content of those volumes, and thus the availability of underlying tools, without the need to modify and rebuild the machine image. This is convenient for end users as they can focus on using the tools while the CloudMan developers can focus on ensuring the infrastructure behaves as desired. Specifically, in order to update a tools data volume, a user instantiates a cloud cluster, modifies the contents of the tools data volume by installing the desired tools (performed just like on any other machine and also showcased by our tool deployment script), creates a snapshot of the modified data volume (done automatically through the web UI), and points their cluster setup to use the newly created data volume snapshot (done manually through a web UI). As a result, the functionality and the architecture of CloudMan can easily be adjusted to the computing needs of individual researchers and different domains. This makes it possible for end users to add their own tools to a CloudMan cluster instance.

## List of abbreviations used

AWS: Amazon Web Services; DNA: Deoxyribonucleic acid; EBS: Elastic Block Storage; EC2: Elastic Compute Cloud; NGS: Next-Generation Sequencing; S3: Simple Storage Service; SGE: Sun Grid Engine; UI: User Interface.

## Competing interests

Authors have no competing interests in this project.

## Authors' contributions

EA, DB, NC, BC, AN, and JT conceived the project, structured the conceptual plans, implemented the software, validated the functionality, and wrote the manuscript. All authors have read and approved the final version of the manuscript.

## References

[B1] Armbrust M Fox A Griffith R Joseph AD Katz R Konwinski A Lee G Patterson D Rabkin A Stoica I Zaharia M Editor ed.^eds. Above the Clouds: A Berkeley View of Cloud Computing Book Above the Clouds: A Berkeley View of Cloud Computing 2009 University of California at Berkeley 23

[B2] Langmead B Schatz MC Lin J Pop M Salzberg SL Searching for SNPs with cloud computing Genome Biol 2009 10 R134 10.1186/gb-2009-10-11-r134 19930550 PMC3091327

[B3] Schatz MC CloudBurst: highly sensitive read mapping with MapReduce Bioinformatics 2009 25 1363 1369 10.1093/bioinformatics/btp236 19357099 PMC2682523

[B4] Wall DP Kudtarkar P Fusaro VA Pivovarov R Patil P Tonellato PJ Cloud computing for comparative genomics BMC Bioinformatics 2010 11 259 20482786 10.1186/1471-2105-11-259 PMC3098063

[B5] Schatz MC Langmead B Salzberg SL Cloud computing and the DNA data race Nat Biotechnol 2010 28 691 693 10.1038/nbt0710-691 20622843 PMC2904649

[B6] The Galaxy Project http://galaxyproject.org/

[B7] Field D Tiwari B Booth T Houten S Swan D Bertrand N Thurston M Open software for biologists: from famine to feast Nat Biotechnol 2006 24 801 803 10.1038/nbt0706-801 16841067

[B8] Taylor J Schenck I Blankenberg D Nekrutenko A Using Galaxy to perform large-scale interactive data analyses Current Protocols in Bioinformatics 2007 19 10.15.11 10.15.25 10.1002/0471250953.bi1005s19 PMC3418382 18428782

[B9] Amazon Elastic Compute Cloud (Amazon EC2) http://aws.amazon.com/ec2/

[B10] Grid Engine http://gridengine.sunsource.net/

[B11] Amazon Elastic Block Store (EBS) https://aws.amazon.com/ebs/

[B12] Cloud Biolinux http://www.cloudbiolinux.com/

[B13] Bio-Linux 6 http://nebc.nerc.ac.uk/tools/bio-linux/bio-linux-6.0

[B14] Langmead B Trapnell C Pop M Salzberg SL Ultrafast and memory-efficient alignment of short DNA sequences to the human genome Genome Biol 2009 10 R25 10.1186/gb-2009-10-3-r25 19261174 PMC2690996

[B15] Li H Durbin R Fast and accurate short read alignment with Burrows-Wheeler transform Bioinformatics 2009 25 1754 1760 10.1093/bioinformatics/btp324 19451168 PMC2705234

[B16] Li H Handsaker B Wysoker A Fennell T Ruan J Homer N Marth G Abecasis G Durbin R The Sequence Alignment/Map format and SAMtools Bioinformatics 2009 25 2078 2079 10.1093/bioinformatics/btp352 19505943 PMC2723002

[B17] Keahey K Freeman T Contextualization: Providing one-click virtual clusters IEEE International Conference on eScience 2008 Indianapolis, IN 301 308 full_text

